# Thermal Reverse-Engineered Synthesis and Catalytic Activity of Nanogold-Containing Silica Aerogels

**DOI:** 10.3390/gels11020087

**Published:** 2025-01-23

**Authors:** Hanna Judit Csupász-Szabó, Boglárka Döncző, Máté Szarka, Lajos Daróczi, István Lázár

**Affiliations:** 1 Department of Inorganic and Analytical Chemistry, University of Debrecen, Egyetem tér 1, 4032 Debrecen, Hungary; szabo.hanna.judit@science.unideb.hu; 2 HUN-REN Institute for Nuclear Research (ATOMKI), Bem tér 18/c, 4026 Debrecen, Hungary; donczo.boglarka@atomki.hu (B.D.); szarka.mate@atomki.hu (M.S.); 3 Department of Solid State Physics, University of Debrecen, Bem tér 18/b, 4026 Debrecen, Hungary; lajos.daroczi@science.unideb.hu

**Keywords:** silica aerogel, nanogold, catalytic activity, immobilization, thermal treatment, nanoislet

## Abstract

Silica aerogels are extensively used as catalyst supports due to their mesoporous structure and chemical inertness. In this study, SiO_2_–AuNP aerogels containing gold nanoparticles (AuNPs) were synthesized using the sol-gel method followed by supercritical CO_2_ drying. The inclusion of polyvinyl pyrrolidone (PVP) as a stabilizing agent preserved the gold particle sizes during the gelation process. In contrast, aerogels synthesized without PVP contained enlarged AuNP aggregates, resulting in a shift in the plasmon resonance color from red to bluish or blue–grey. Thermal treatment of these bluish-colored aerogels at high temperatures restored their red coloration, visually indicating the breakdown of large gold clusters into individual nanoparticles. Both types of aerogels were characterized using SEM, TEM, 3D optical microscopy, UV–vis and ATR-IR spectroscopy, and N_2_ porosimetry, with their properties analyzed as a function of annealing temperature. Their catalytic activity was evaluated through the reduction of 4-nitrophenol with sodium borohydride, and both aerogel types demonstrated catalytic activity. This thermal conversion of large clusters into individual nanoparticles within an aerogel matrix introduces a new and promising approach for creating catalytically active nanogold-containing aerogel catalysts.

## 1. Introduction

Nanomaterials and nanoparticles are at the forefront of modern science and technology, representing a rapidly expanding field of research with numerous practical applications. Metal nanoparticles are particularly significant due to their unique optical, physical, and chemical properties compared to their bulk counterparts [1,2].

Gold nanoparticles, in particular, have garnered significant interest for both theoretical and practical reasons, as their size and shape can be precisely controlled to form spheres [3,4], rods [5,6], cubes [7], and stars [8], allowing for extensive study [9,10]. Their applications span medical [11], biological [12], catalytic [13], optical, and sensor technologies [14]. Colloidal gold nanoparticles are typically synthesized using the Turkevich method, a bottom-up approach where chloroauric acid is reduced and stabilized by sodium citrate. This method is renowned for its simplicity and effectiveness, producing gold nanoparticles with a diameter of approximately 10 nm [15].

Aerogels are among the most intriguing examples of nanostructured porous solids, characterized by their extremely low density (0.004–0.500 g/cm^3^) and high specific surface area. Their mesoporous nanostructure makes them highly functionalizable, enabling a broad range of applications. Aerogels are increasingly used as heat and sound insulators [16,17], components in cosmetics [18], materials for controlled drug delivery [19], catalysts or catalyst carriers [20,21], and in advanced fields such as nuclear physics and the aerospace industry [22].

Due to their highly porous structure, inorganic aerogels are well-suited for forming nanocomposites with metal nanoparticles, alloys, and metal oxides [23,24,25]. The distribution and integration of nanoparticles into the gel matrix can be achieved through several methods. One approach involves adding nanoparticles to the reaction mixture during sol-gel synthesis [26]. This method allows for even nanoparticle distribution and precise control of their quantity. Another technique introduces nanoparticles into the aerogel structure by soaking the wet gel in a nanoparticle solution [27], resulting in the formation of a depth gradient. By incorporating nanoparticles—either covalently attached to or embedded in the matrix—aerogels gain enhanced properties and functionalities, significantly broadening their range of applications. Silica or alumina aerogels or aerogel-like materials containing gold nanoparticles have gained remarkable attention most recently due to their special optical [28,29], plasmonic [30], catalytic and biological (i.e., antibacterial) [31] activities. Furthermore, gold nanoparticles can not only act as guest particles in aerogel matrices but are also capable of forming gold aerogels on their own, showing enhanced catalytic activity [32].

**Figure 1:**
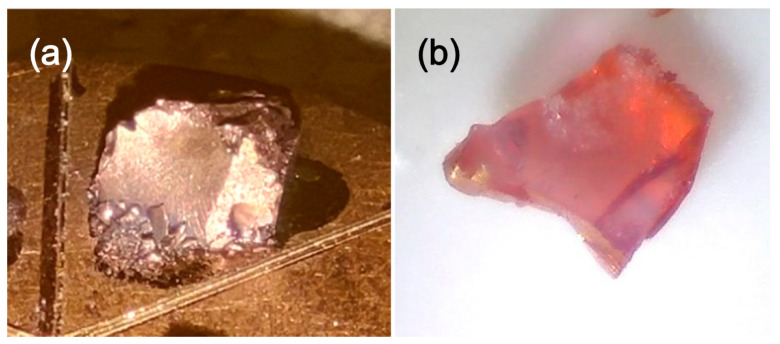
Thermal change of sputtered gold layer after heating at 500 °C for 8 h. (**a**) Gold-sputtered silica aerogel sample on a SEM sample holder; (**b**) the same specimen after heat treatment. The loss of metallic luster and the appearance of the red color is a clear indication of the formation of plasmonic gold nanoparticles.

In a prior investigation, we successfully immobilized gold nanoparticles in silica aerogels. However, the stability of citrate-stabilized gold nanoparticles was compromised during aerogel synthesis, leading to rapid aggregation during the sol-gel process. This challenge was addressed by employing PVP as a stabilizing agent, enabling the reproducible synthesis of multi-centimeter-sized monolithic silica aerogel–gold nanocomposites without aggregation of the nanogold guest particles [33]. Despite this progress, a notable number of bluish or blue–grey-colored, aggregated, gold-containing samples were also generated in the course of our development efforts. Inspired by our observations on the high-temperature thermal behavior of a gold-sputtered silica aerogel sample, as shown in Figure 1, and the formation of plasmonic gold nanoparticles from the surface gold nanolayer, we considered whether this behavior could be harnessed to generate (reverse-engineer) catalytically active red-colored gold nanoparticles from the large AuNP aggregates present in catalytically nearly inactive blue–grey aerogels. In addition to restoring catalytic activity, we systematically investigated the thermal transformation of a gold layer or gold nanoparticle aggregates deposited on the surface or embedded within the aerogel matrix.

The reduction of 4-nitrophenol (4-NP) with sodium tetrahydroborate (NaBH_4_) was chosen as the reaction for catalytic tests due to the feasibility and simplicity of investigations, the lack of side reactions, and, most importantly, the high potential and value of such catalysts in environment protection. 4-nitrophenol ranks 58th on the EPA’s list of persistent pollutants [34]. The reduction of 4-NP to 4-aminophenol (4-AP) with sodium borohydride in the presence of noble metal nanoparticles [35,36] has been extensively studied [37,38,39]. Most recently, gold nanoparticles immobilized on different solid carriers like mesoporous silicas, cellulose beads, alginate-modified magnetic particles, or mesostructured oxides were used for the reduction of 4-NP, as well as environmentally dangerous organic dyes and methylene blue [40,41,42,43]. Without immobilization, nanoparticle release would be the source of secondary environmental contamination [44]. Therefore, the development of an easily removable and regenerable heterogeneous catalyst is crucial.

## 2. Results and Discussion

### 2.1. Thermal Behavior of Sputtered Gold Layers on Silica Aerogel Surfaces

Small monolithic silica aerogel cylinders (pre-treated at 500 °C for 5 h) were sputtered with gold to deposit an approximately 15 nm thick layer on the surface. After sputtering, the samples were heated in a muffle furnace at 500 °C and 900 °C for 1 h.

**Figure 2:**
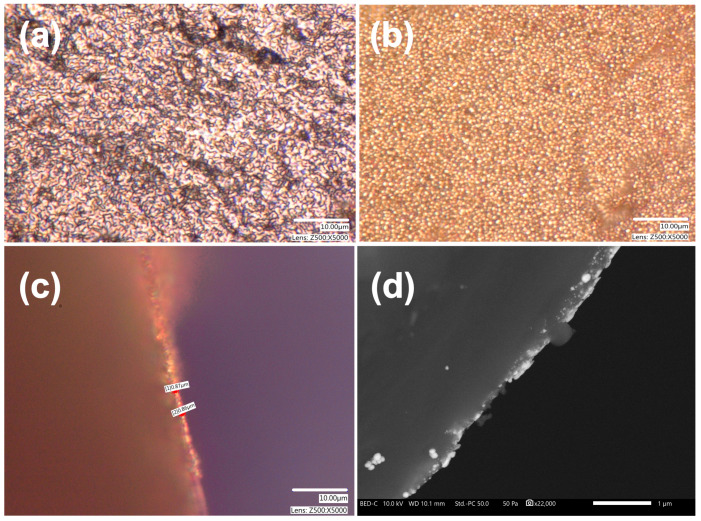
Optical microscopy images of (**a**) 15 nm thick sputtered gold layer on silica aerogel, (**b**) gold islands formed on the silica surface when heated to 900 °C, (**c**) gold islets on the surface and in-depth migration/formation of gold particles in the silica matrix. The depth of penetration is approx. 1 μm, significantly exceeding the thickness of the sputtered gold layer (scale bars: 10 μm). (**d**) Cross-sectional scanning electron microscopy image of different-sized gold nanoparticles on the surface and in the outer 200 nm layer of the silica aerogel. Scale bar: 1 μm.

High-power 3D optical microscopy and scanning electron microscopy were employed to study the structural changes. The analysis revealed that the sputtered gold film underwent gradual decomposition and migration, and gold islets were formed on the surface and inside the outermost 500–900 nm layer of the porous silica structure (Figure 2).

Figure 2a shows the original sputtered surface layer, which covers the globular structure of the silica aerogel. Increasing the temperature results in a migration of gold atoms and formation of larger gold islets.

The islet formation became the dominant feature at 900 °C, as shown in Figure 2b. A cross-sectional view of the surface layer revealed an approximately 1 μm deep penetration of gold into the matrix. However, this value is rather inaccurate due to the high transparency of the silica matrix and the light scattering on gold particles in the deeper layers, as shown in Figure 2c. The scanning electron microscopy image in Figure 2d provides more accurate information on the depth of penetration and the shape of the gold nanoparticles. A cylindrical sample was cut in half and polished on diamond lapping paper before the study. The image in Figure 2d shows diffuse penetration of the gold into the silica aerogel and the formation of smaller, nanometer-sized and larger nanoparticles of up to 40 nm size inside. This is in good agreement with the migration and transformation of in situ-generated gold thin film in mesoporous silicas like MCM-41 and SBF-15 [45].

**Figure 3:**
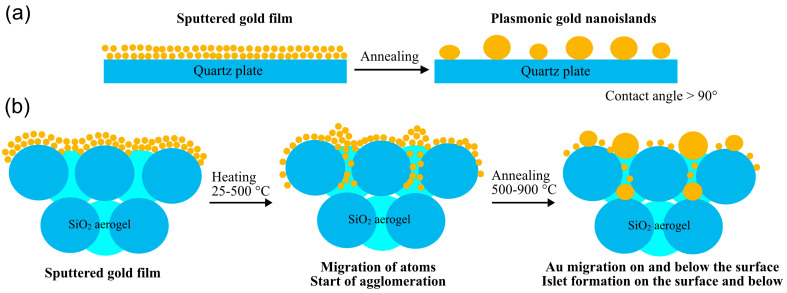
(**a**) Demonstration of gold islet formation from sputtered gold layer at a high temperature on a quartz surface. (**b**) Proposed mechanism of the migration and transformation of a sputtered gold layer on silica aerogel forming gold nanoparticles and islets on the surface and inside the aerogel matrix upon high temperature heat treatment.

Gold nano-islet formation on substrates like quartz, glass, silicon, or graphite has been extensively studied in the literature, leading to the conclusion that the growth of AuNPs from thin films can be described by the kinetic aggregation of gold atoms and smaller clusters and not by Ostwald ripening [46]. Thermal annealing of thin gold films on such smooth substrates leads to the formation of gold nanoislets, as is demonstrated in Figure 3a. Their size and morphology strongly depend on the material and surface property of the substrate, the film thickness, the chemical composition of the annealing atmosphere, the temperature, and time [47,48,49].

Mesoporous monolithic silica aerogel surfaces, however, have not been used as a substrate for the generation of plasmonic gold nanoparticles before, although gold sputtering of such surfaces is a standard process to provide electrically conductive surfaces for transmission electron microscopy studies. To summarize the transformations of the surface gold layer into surface-bound and internally generated gold nanoparticles, we propose the mechanism depicted in Figure 3b. Migration of the gold atoms occurs even at temperatures as low as 175 °C [46], but typically, the 300–450 °C range is used for the generation of gold nanoparticles. In our experiments, the lowest temperature was 500 °C, where the rapid migration of the surface gold layer and its coalescence into larger particles was observed, which became dominant at 900 °C. Most likely, the migration of gold had started at temperatures lower than 500 °C, but this range was out of the scope of the present study.

### 2.2. Thermal Behavior of Dispersed Gold Nanoparticles in Silica Aerogels

**Figure 4:**
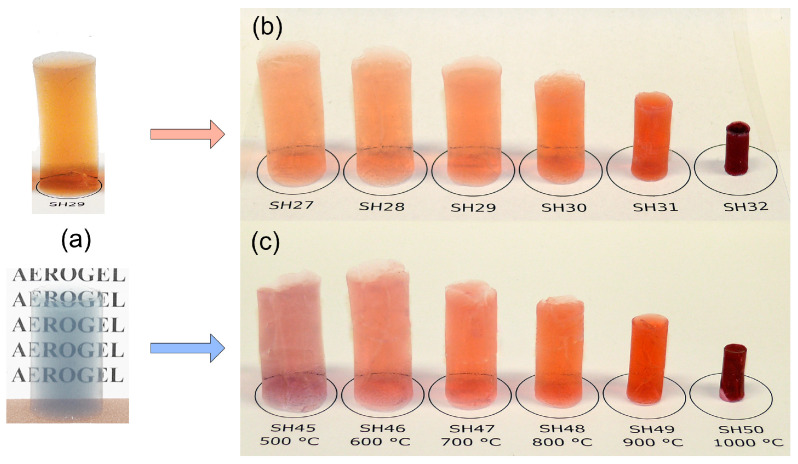
(**a**) PVP-stabilized and non-stabilized (bluish-colored) gold nanoparticle-containing aerogels after supercritical drying at 25 °C. (**b**) PVP-stabilized gold nanoparticle-containing aerogels after high-temperature heat treatment, and (**c**) non-stabilized gold nanoparticle-containing aerogels.

Two series of monolithic silica aerogels containing nanogold particles, both with and without PVP stabilizers, were annealed simultaneously at temperatures of 500 °C (SH45, SH27), 600 °C (SH46, SH28), 700 °C (SH47, SH29), 800 °C (SH48, SH30), 900 °C (SH49, SH31), and 1000 °C (SH50, SH32). For PVP-stabilized nanogold-containing aerogels, no significant color change was observed; only a variation in intensity, indicating that the size of the nanoparticles remained virtually unaltered. For aerogel samples containing non-stabilized gold nanoparticles, a pronounced color change was observed during thermal treatment (Figure 4). The change from blue–grey to the red color shown in Figure 4a,c is a visible and obvious indication of the transformation of large nanogold aggregates into smaller-sized gold nanoparticles of approximately the same size that are present in the simultaneous counterparts shown in Figure 4b.

**Figure 5:**
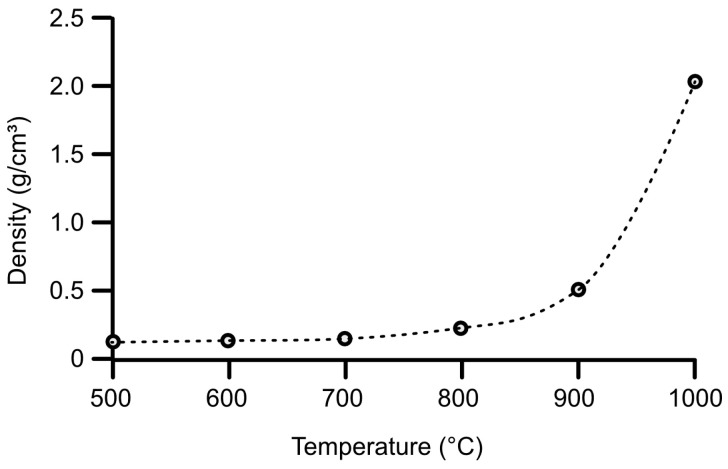
Apparent density of PVP-stabilized AuNP-silica aerogels vs. the annealing temperature.

As shown in Figure 4, the silica matrix underwent progressive shrinkage with increasing temperature, leading to a substantial reduction in volume. This was due to water loss in high-temperature condensation reactions in which new Si-O-Si bonds were formed. At temperatures above 900 °C, the slow viscous flow of the amorphous silica globuli resulted in a decreased surface area. Consequently, as depicted in Figure 5, the apparent density of the aerogels exhibited a rapid increase at elevated temperatures.

The specific surface area and pore size of the annealed aerogels were assessed using nitrogen adsorption–desorption porosimetry, with the results summarized in Table 1. Thermal treatment significantly reduced the specific surface area as the annealing temperature increased. The typical pore size was around 20 nm, except for aerogels annealed at 1000 °C (SH32, SH50), where smaller pores resulted from the extreme shrinkage of the silica matrix. PVP as a stabilizing agent contributed to larger pore sizes and greater shrinkage during heating, influencing the structural properties of the aerogels.

**Figure 6:**
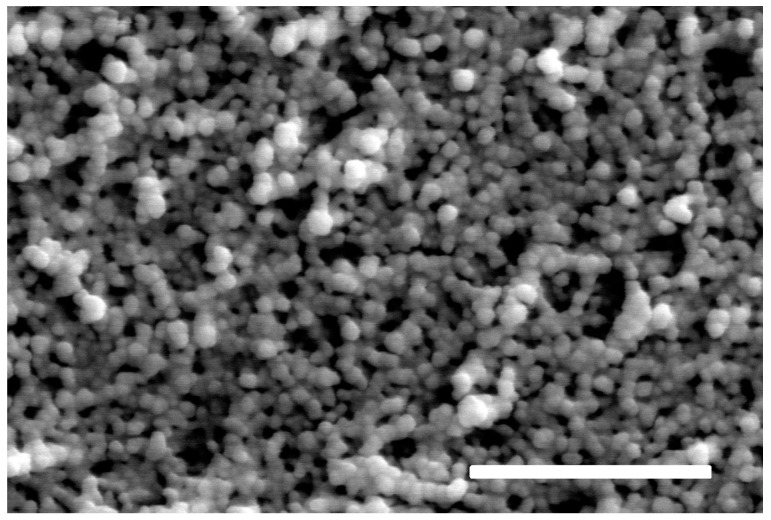
SEM picture of the fine globular structure of an aggregated gold nanoparticle-containing bluish monolithic silica aerogel shown in Figure 4a. Scale bar: 1 μm.

The as-prepared aerogels were characterized using electron microscopy techniques (SEM and TEM). SEM images confirmed that the SiO_2_ matrix and pore structure were homogeneous across all samples (Figure 6). However, gold nanoparticles could not be distinguished in the images due to their nearly identical shape and size to the silica globules.

**Figure 7:**
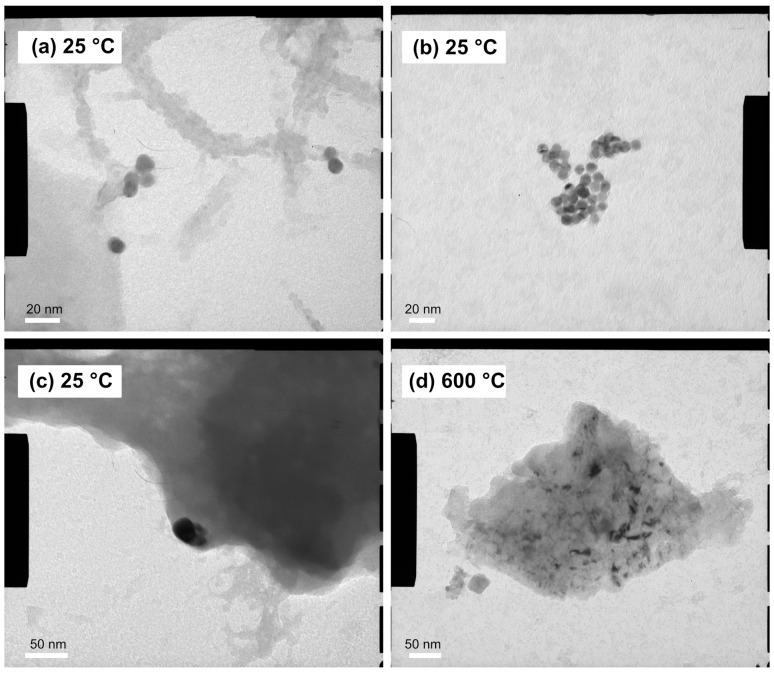
Transmission electron microscopy images of solution-phase and aerogel-embedded gold nanoparticles. (**a**) Spherical citrate-stabilized gold nanoparticles in colloidal form, (**b**) aggregated nanogold particles formed in solution, (**c**) blue–gray-colored aggregated gold nanoparticle embedded in the silica aerogel matrix, (**d**) annealed gold nanoparticles in the range of 4–20 nm diameter dispersed/generated in the silica aerogel matrix.

TEM images provided detailed information on the size and shape of gold nanoparticles in colloidal solutions, both in stabilized and non-stabilized states, as well as in aggregated and re-dispersed forms embedded within the aerogels (Figure 7). The PVP-stabilized nanoparticles were approximately 12–14 nm in diameter and exhibited only weak associations with one another (Figure 7a). In contrast, the nanoparticles in unstabilized (blue-colored) nanogold solutions formed large, strongly connected aggregates. These solutions were used to prepare the monolithic aerogel composites shown in Figure 4a. In these composites, while individual nanoparticles were evenly distributed within the silica matrix, large aggregates were densely packed and surrounded by covalently bound silica globules, as shown in Figure 7c.

The structural changes in the unstabilized aerogel composite upon annealing at 600 °C were examined using transmission electron microscopy. These changes are attributed to the shrinkage of the aerogel matrix, creating a “squeezing” effect, and the subsequent release of loosely connected outer nanoparticles. Melting point suppression, a well-documented phenomenon for nanometer-sized particles, may also contribute to the changes [50]. Due to gold’s relatively low Tammann temperature (395 °C), heating induces sintering of gold clusters, significantly increasing the mobility of the metal particles [51]. At elevated temperatures, the ongoing shrinkage of the silica matrix likely compresses the aggregated gold particles, leading to their dispersion and an apparent size reduction. Additionally, significant structural changes may arise from the migration of gold atoms within the pores and the formation of new gold nanoparticles at a distance from the original aggregates. This phenomenon is similar to that observed in the sputtered gold layers described above. The presence of 4–20 nm sized nanoparticles is clearly shown in Figure 7d.

**Figure 8:**
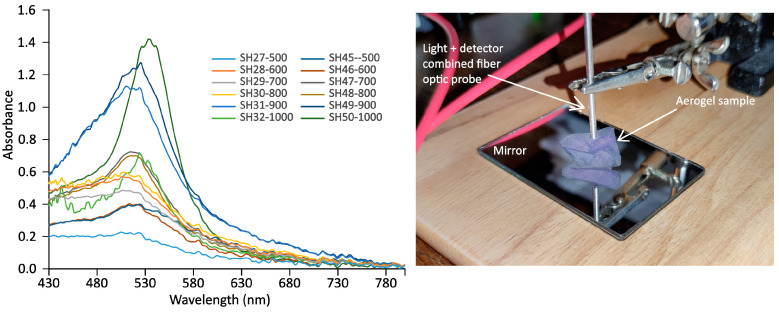
Visible spectra of stabilized and unstabilized nanogold particle-containing aerogels after 500–1000 °C annealing. The picture on the right side shows the mirror reflection arrangement of the fiber optic probe over an irregular-shaped aerogel sample. Due to the large differences and shapes of the specimens, absorbance values cannot be compared.

**Figure 9:**
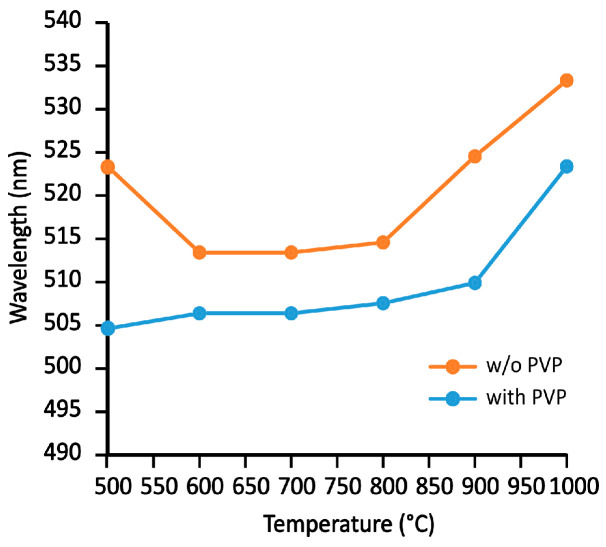
Change in the wavelength of peak maxima on the visible spectra of annealed aerogel samples shown in Figure 4b,c.

UV–vis spectroscopy is a widely used method for analyzing the size, shape, and concentration of gold nanoparticles, as their size- and shape-dependent characteristic light absorption occurs in the 400–900 nm range [52]. Spherical particles exhibit a single absorption maximum in the visible spectrum. The visible spectra of solid aerogel samples were recorded using a fiber optic spectrometer with a combined reflection probe. To enhance sensitivity, a flat mirror was incorporated into the setup (Figure 8), effectively doubling the light path. While this technique theoretically allows for concentration determination, the fractured and mechanically fragile nature of the samples made determining their shapes for quantitation impractical. However, the temperature dependence of the spectral maxima, as shown in Figure 9, provided valuable insights into the behavior of the gold nanoparticles in the annealing process. The surface plasmon resonance (SPR) maximum wavelength values are in strong connection with the size of the gold nanoparticles, as described in the literature [53,54,55]. Thus, it was possible to follow the change in particle sizes as a function of the temperature. Characteristic wavelengths can be attributed not only to embedded nanoparticles but also to the surface nanoislets, and this can be used to determine their sizes [56].

The series of unstabilized aerogels (Figure 9, orange line) follows the expected trend in the 600–800 °C range, while a decrease in the maximum from 500 °C to 600 °C corresponds to the size reduction of large gold aggregates. The particle sizes remained relatively stable until the temperature reached 900 °C and 1000 °C, at which point a significant increase was observed. This was likely due to the formation of larger gold nanoparticles in the voids resulting from suppressed melting and increased migration/diffusion of the gold. This behavior aligns with the melting point of approximately 950 °C for 10 nm gold particles published in the literature [57]. In contrast, the series of PVP-stabilized gold particles (blue line) shows a nearly constant value, indicating that the nanoparticle sizes remained unchanged up to 900 °C. However, at 1000 °C, a similar increase in particle size was observed, consistent with the behavior seen in the unstabilized aerogels.

The absolute wavelength values for both series are unexpectedly low. Such low values have not been observed in solution studies. Thus far, the lowest SPR value was approximately 518 nm, measured in aqueous solutions. In our case, the approximately 10–15 nm systematic drop cannot be attributed to the presence of extremely small gold nanoparticles because this would contradict the SEM and TEM results. Such low wavelengths values are more likely due to the loss of the soft hydration sphere and the hard electronic environment of the particles.

**Figure 10:**
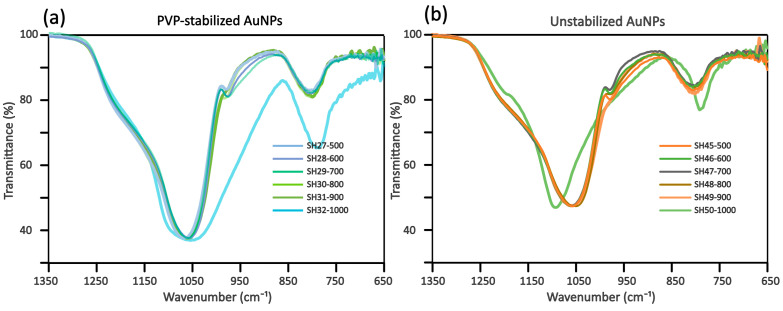
Normalized ATR-IR spectra of nanogold-containing aerogels annealed at different temperatures. (**a**) Spectra of PVP-stabilized gold-containing aerogels, (**b**) spectra of unstabilized nanogold-containing aerogels.

ATR-IR analysis of the annealed aerogel composites indicated that the aerogel structure and degree of hydration remained virtually unchanged within the 500–900 °C range. The only noticeable difference in the spectra (Figure 10) was a shift in the Si-O-Si vibrations for the 1000 °C samples, attributed to extensive condensation and water loss from residual silanol groups. However, the presence or state of gold nanoparticles could not be confirmed using this technique due to their very low concentrations relative to the weight of the silica matrix.

**Figure 11:**
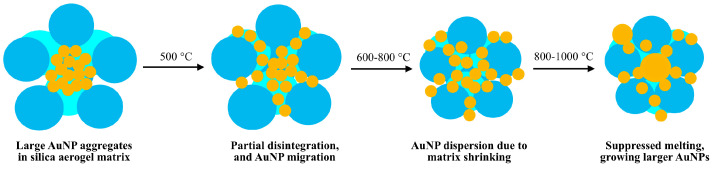
Proposed mechanism of thermal disintegration and dispersion of Au nanoparticle aggregates followed by their coalescence into gold globules in a silica aerogel matrix in the 500–1000 °C temperature range.

Figure 11 summarizes the proposed mechanism of the transformation of large gold nanoparticle aggregates into smaller nanoparticles, and then the reversal of the particle sizes upon very-high-temperature annealing due to the suppressed melting, migration, and coalescence of the gold nanoparticles, forming gold globules inside the shrinking and not-wettable silica matrix. Their formation was not reflected by the deep-red color of the 1000 °C aerogels, and this revealed only by the place of plasmon resonance maximums in the UV–vis spectrum (Figure 9).

### 2.3. Catalytic Activity

Catalytic reduction experiments were performed using colloidal gold nanoparticles, PVP-stabilized gold nanoparticle-containing aerogels, and reverse-engineered nanoparticle-containing aerogels.

**Figure 12:**
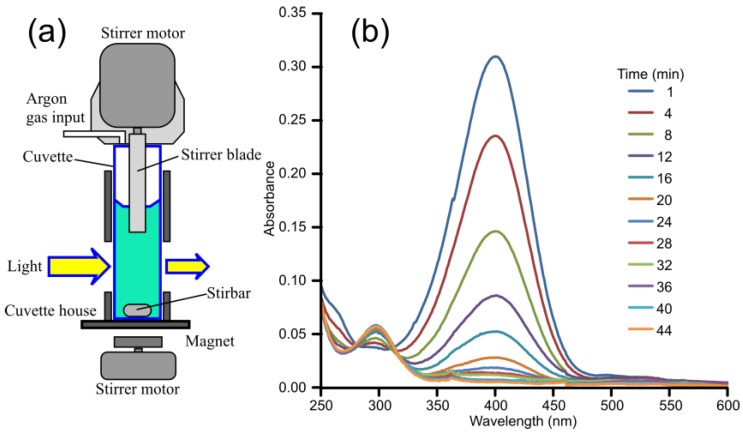
(**a**) The equipment used to carry out the experiment in a cuvette, providing continuous stirring under an argon gas atmosphere. (**b**) Time-dependent absorption spectrum of 4-NP reduced by a large excess of NaBH_4_ in the presence of 10 nm gold nanoparticles.

Due to the initial irreproducibility of the kinetic measurements carried out in simple quartz cuvettes, not only the catalyst’s quality but other factors influencing the reduction process were considered. Such factors were the mode and rate of stirring (Figure 12a, upper, lower), the material of the stirring blades (ptfe, stainless steel, nickel), and the presence or absence of reactive atmospheric gases (CO_2_, O_2_). The stirring rate played a minor role only in removing hydrogen bubbles from the light path, and other factors (other than O_2_) had no impact on the results. Consistent with the findings of Menumerov et al. [58], we observed that in the presence of gold nanoparticles, atmospheric oxygen inhibited the 4-NP to 4-AP reduction reaction. To address this, the reaction was conducted under an argon atmosphere, and the solutions were deoxygenated beforehand by bubbling argon gas through them. Magnetic stirring ensured the even distribution of reagents and the catalyst within a cuvette during the reaction (Figure 12a).

When NaBH_4_ was added to the aqueous solution of 4-nitrophenol, 4-nitrophenolate ions formed immediately, producing a bright-yellow solution due to the pH change. In the presence of a catalyst, the yellow color gradually faded as the reduction progressed. This process was easily monitored using UV–vis spectroscopy. The reaction’s progress and conversion rate were tracked by observing the decrease in the spectral peak at 400 nm, corresponding to 4-nitrophenolate ions, and the simultaneous appearance of a peak at 300 nm, indicating the formation of 4-aminophenol (Figure 12b). Without a catalyst, the reduction of 4-nitrophenolate ions was negligible, demonstrating that the transformation into 4-aminophenol did not occur under such conditions.

**Figure 13:**
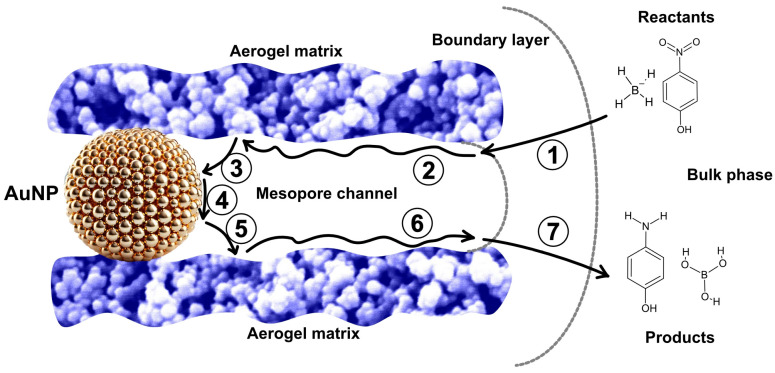
Main steps of the heterogeneous catalytic process of the reduction of 4-NP to 4-aminophenol. (1) Film diffusion of the reactants from the bulk phase through the boundary layer to the surface, (2) mesopore diffusion, (3) adsorption on AuNP, (4) catalytic reaction, (5) desorption of the products, (6) mesopore diffusion, (7) film diffusion through the boundary layer into the bulk phase.

The reduction of 4-NP was catalyzed by gold nanoparticle-containing aerogels, with the nanoparticles located within the catalyst’s pores. For the reaction to proceed, the reactant molecules must reach the active sites. This type of heterogeneous catalysis involves a sequence of seven main steps (Figure 13).

Initially, the reactants diffuse from the bulk phase to the external surface of the solid phase (film diffusion). Next, they enter the mesopores and migrate to the vicinity of the catalytic surface (mesopore diffusion), where they are adsorbed as the third step. The reaction then occurs at the active sites. The products formed are subsequently desorbed and diffuse out from the pore interior to the catalyst’s external surface. Finally, the product diffuses from the external surface into the bulk phase, completing the catalytic cycle [59,60].

According to the literature, the reduction of 4-NP can be described by the Langmuir–Hinshelwood mechanism, which involves multiple steps. The key aspect of this mechanism is that both reactants must be adsorbed onto the catalyst’s surface for the reaction to proceed. In this process, 4-NP is reduced to 4-aminophenol by surface-hydrogen species generated through the hydrolysis of BH_4_− ions. The diffusion, adsorption, and desorption steps are assumed to be rapid and reversible, making the reduction of 4-NP the rate-determining step [39,61,62].

For reverse-engineered nanoparticles in aerogels, two different amounts of catalysts were tested for each batch and sample. Initially, 84 mg (84.0 ± 0.3 mg) of annealed aerogel samples (500–900 °C) were used to study the reduction. Among these, the aerogel annealed at 500 °C demonstrated the highest conversion rate (0.72), outperforming the other samples. Aerogels annealed at 600 °C, 700 °C, and 800 °C showed comparable conversion rates, with no significant differences. The aerogel annealed at 900 °C exhibited the lowest conversion rate, attributed to the reduced accessibility of the reactants to the gold nanoparticles due to the substantial shrinkage of the silica matrix.

Since no significant differences in catalytic activity or conversion were observed among the samples annealed at 600–800 °C, the catalyst amount was reduced to 60 mg for subsequent reactions.

**Figure 14:**
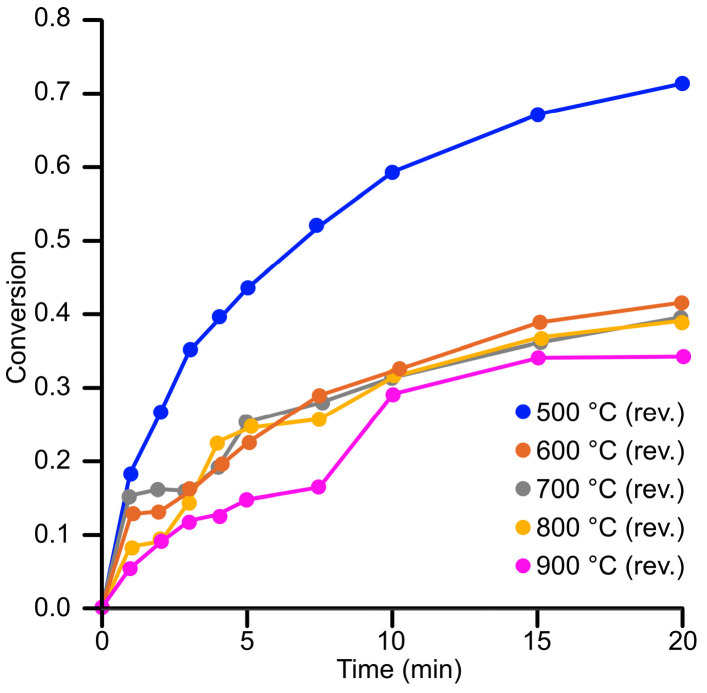
Catalytic activity of reverse-engineered samples annealed at different temperatures in the reaction of the reduction of 4-NP.

**Figure 15:**
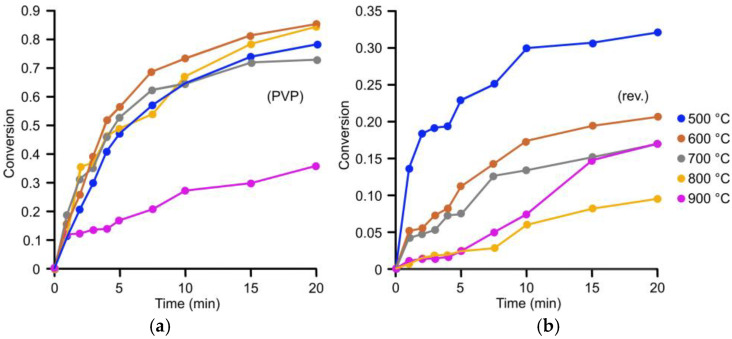
Conversions of the catalytic reduction applying 60 mg of (**a**) PVP-containing and (**b**) reverse-engineered samples annealed at different temperatures.

As shown in Figure 14 and Figure 15, the increase in time-dependent conversion is inconsistent across cases, which can be attributed to challenges in the internal diffusion steps of heterogeneous catalysis. For reverse-engineered aerogel catalysts, a clear trend was observed: higher annealing temperatures resulted in lower conversion rates. This is explained by the progressive shrinkage of the matrix pores with increasing temperature, reducing the accessibility of active sites to reactants.

An exception was noted for the sample annealed at 900 °C, which showed a higher conversion rate than the 700 °C sample. This can be attributed to the heat-induced concentration of gold nanoparticles in the matrix. In contrast, the PVP-stabilized gold nanoparticle-containing aerogels showed relatively consistent conversion rates for samples annealed between 500 and 800 °C. The sample annealed at 900 °C displayed reduced catalytic activity due to limited access to the catalytic sites.

Overall, the PVP-stabilized nanoparticle-containing aerogels provided higher conversions than their reverse-engineered counterparts. This aligns with the nitrogen porosimetry results (Table 1), which suggest that better accessibility to active sites in the PVP-stabilized samples contributes to their superior catalytic performance.

The reaction followed a first-order rate law with respect to the concentration of 4-NP, using borohydride in excess. A directly linear correlation was observed when plotting the ln(At/A0) values against time [38]. The apparent rate constants were determined using MicroMath Scientist 2.01 software, where applicable (Table 2). The rate constants (k) and corresponding half-lives (t_1/2_) of the reactions were calculated for the colloidal gold nanoparticles and aerogels used as catalysts, provided the correlation coefficient exceeded 0.95. The half-life values were derived using Equation (1).

t_1/2_ = ln(2)/k (1)

When comparing the PVP-containing samples with the reverse-engineered samples annealed at 500 °C, it is evident that, despite using a greater amount of catalyst, the reaction rate is lower, and the half-life is longer for the reverse-engineered sample.

**Figure 16:**
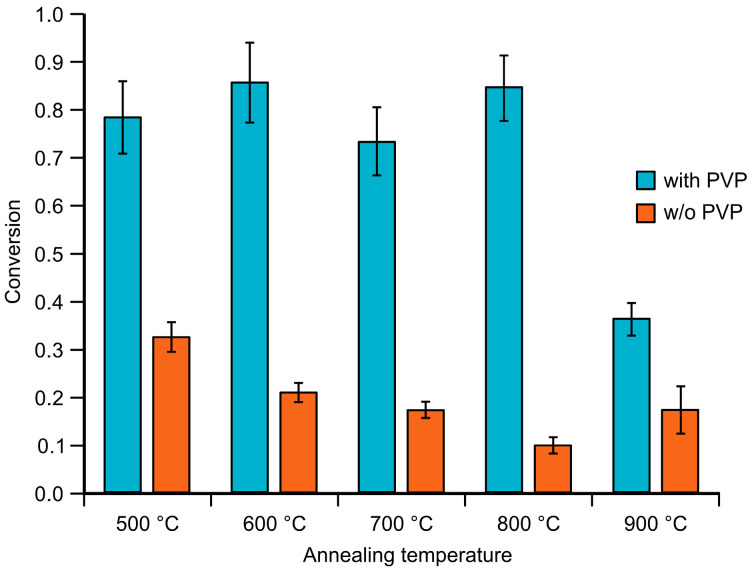
Conversions achieved after 20 min of the catalytic reduction of 4-NP using 60 mg of heat-treated PVP-stabilized and reverse-engineered aerogel catalysts.

Conversions achieved at 20 min were plotted against annealing temperatures (Figure 16), clearly showing that the PVP-stabilized gold nanoparticles provided a more efficient catalytic reaction compared to their reverse-engineered counterparts.

In summary, two key factors must be considered when evaluating the catalytic reactions of gold nanoparticle-containing aerogels. First, PVP influences the aerogel’s fine structure by enlarging the matrix pores, facilitating easier access for reagents to reach the catalytically active gold nanoparticles. Second, the annealing process causes matrix shrinkage, reducing pore accessibility and limiting the interaction between reagents and nanoparticles. Although matrix shrinkage increases the concentration of nanoparticles in a given volume, this effect is insufficient to offset the reduced accessibility caused by the decreased pore size.

Gray-colored, non-stabilized gold nanoparticle-containing aerogels, even without the annealing process, exhibited catalytic activity, achieving a conversion of 0.50 at 20 min. This performance surpassed that of the non-stabilized annealed samples. These results indicate that while the particle sizes, exhibited by the change in the surface plasmon resonance wavelength, can be reverse-engineered, the shrinkage of the silica matrix plays a more critical role in determining catalytic efficiency due to its impact on the accessibility of active sites.

The conversion achieved with gold colloidal nanoparticles (0.91 at 20 min) was higher than that of immobilized particles at any annealing temperature. This difference in apparent catalytic activity aligns with expectations, as homogeneous phase reactions allow for easier interactions between the substrate and catalytic particles, whereas in heterogeneous systems, encapsulation within the aerogel network inhibits such interactions.

## 3. Conclusions

We have developed an innovative process for controlling the particle size of gold nanoparticles embedded in silica aerogels through thermal treatment. Large aggregates of gold nanoparticles, which exhibit moderate catalytic activity, can be reactivated at higher temperatures by dispersing them into smaller, more active particles—a process referred to as reverse-engineering.

Our study demonstrated that both PVP-stabilized and reverse-engineered nanogold-containing aerogels are effective catalysts for the reduction of the environmentally hazardous pollutant 4-nitrophenol. The findings show that even larger aggregates embedded within a silica aerogel matrix can be transformed into smaller, catalytically more active nanoparticles. PVP-stabilized gold nanoparticle-containing aerogels are more advantageous for catalytic applications than reverse-engineered nanoparticle-containing aerogels owing to their structural integrity and better catalytic performance. However, reverse-engineered AuNP-silica aerogels, while exhibiting slightly lower activity, are valuable catalyst materials due to their simpler and less-sensitive preparation process.

These understandings highlight the importance of thermal reverse engineering. By applying high-temperature treatment, catalytically nearly inactive gold aggregates in silica aerogels can be converted into lower-nanoscale particles, leading to a novel method: catalytic re-activation via annealing. This approach provides a technologically distinct yet straightforward solution for particle size control. In addition, the confined-space thermal approach provides a promising new method for producing metal nanoparticle-containing silica or other inorganic oxide aerogels, even when nanoparticle aggregation is unavoidable during the gelation process.

## 4. Materials and Methods

### 4.1. Materials

The following chemicals and reagents were purchased and used without further purification: nanogold solution (10 nm) stabilized with citrate (Sigma-Aldrich, St. Louis, MO, USA), ammonia solution of 25% (m/m) (Molar Chemicals, Halásztelek, Hungary), tetramethoxysilane (TMOS) (Fluka, St. Gallen, Switzerland), methanol (technical grade, Molar Chemicals), methanol (HPLC grade, Sigma), acetone (99.9%, Molar Chemicals), and poly(vinylpyrrolidone) (MW 40kD, Sigma-Aldrich).

UV–vis spectrophotometry studies were performed with a Metertech SP-8001 (Metertech Inc., Nangang, Taipei, Taiwan) spectrophotometer in the 250–800 nm range, using quartz or disposable PMMA cuvettes, depending on the spectral range. Data were registered and analyzed using the Metertech UV-Mate software (Metertech Inc., Nangang, Taipei, Taiwan). Visible spectra of solid aerogel samples were recorded using an Avantes fiber optic spectrophotometer in a mirror-reflection arrangement. Porosities were measured using a Quantachrome NOVA 2200e instrument (Quantachrome Instruments, Inc., Boynton Beach, FL, USA) and analyzed with the NovaWin 11.0 software (Quantachrome Instruments, Inc., USA). Imaging of the samples was performed using a JEOL JSM-IT500HR-type Scanning electron microscope (JEOL, Tokyo, Japan). Image acquisition was carried out in low vacuum mode (50 Pa N_2_) with an accelerating voltage of 10 kV, probe current value Std. 50, and a working distance of 10 mm. Images were recorded using a backscattered electron detector at various magnifications. The size and shape of the gold nanoparticles in the PVP-stabilized and non-stabilized solutions and aerogels were examined using a JEOL 2000 FX-II electron microscope (JEOL, Tokyo, Japan). Digital 3D microscopic images of the samples were recorded using a Keyence VHX-6000 digital 3D microscope (Keyence, Osaka, Japan) with reflective illumination. UV–vis spectra were recorded using a fiber optic Avantes AvaSpec DAD spectrometer equipped with an AvaLight-DHc light source (Avantes BV, Apeldoorn, The Netherlands). IR spectra were collected using a Agilent Technologies Cary 660 spectrometer (Agilent Technologies, Santa Clara, CA, USA) in ATR mode and processed using the MicroLab 5.6 PC software (Agilent Technologies). Gold contents were determined using an Agilent ICP-OES 5110 spectrometer (Agilent Technologies, Santa Clara, CA, USA).

### 4.2. Synthesis of Aerogels

Alcogels were prepared using the sol-gel method, as detailed below. Reaction mixtures were poured into plastic molds, aged, and solvent-exchanged after placing them in perforated aluminum drying frames. Solvent extractions and supercritical CO_2_ drying of alcogels to aerogels were performed in a high-pressure reactor according to the process described in the literature [63].

#### 4.2.1. Non-Stabilized Nanogold-Containing Aerogels

Using a magnetic stirrer, 18.0 mL of methanol was added to 4.50 mL of citrate-stabilized 10 nm AuNP solution. Then, 9.00 mL of a 1:1 (*v*:*v*) mixture of methanol and TMOS was added to the reaction mixture. Lastly, 4.05 mL of 1:10 (*v*:*v*) diluted 25% (m/m) ammonia solution was added as a catalyst. After 30 s of intensive stirring, the mixture was poured into a 28 mm diameter cylindrical PVC plastic mold and covered with a double layer of Parafilm to prevent evaporation. After one day of aging in the mold, the alcogel was transferred into a pitted aluminum frame and soaked in consecutive steps in methanol, methanol–acetone mixture, acetone, and freshly distilled dry acetone for three days to remove the water residue from the gel. Afterward, the wet gels were transferred into a high-pressure reactor and dried under supercritical conditions.

#### 4.2.2. PVP-Stabilized Nanogold-Containing Aerogels

An aqueous stock solution of polyvinyl pyrrolidone was prepared by dissolving 500 mg of PVP in 10 mL of H_2_O. Using a magnetic stirrer, 3.60 mL of PVP stock solution was added to 4.50 mL of citrate-stabilized 10 nm AuNP solution. After mixing for 5 min, 18.00 mL of methanol and 9.00 mL of a 1:1 (*v*:*v*) mixture of methanol and TMOS were added to the reaction mixture. Lastly, 4.05 mL of 1:10 (*v*:*v*) diluted 25% (m/m) ammonia solution was used as a catalyst. After 30 s of intensive stirring, the mixture was poured into a 28 mm diameter cylindrical PVC plastic mold and covered with a double layer of Parafilm to prevent evaporation. Solvent exchange and drying followed the same process described above.

### 4.3. Annealing Process

To modify and control the aggregation that occurs during the cross-linking of the silica matrix, aerogels with and without the stabilizer (PVP40) were submitted to thermal treatment. After supercritical drying, all aerogel monoliths were heat-treated at 500 °C for 5 h, then selected samples were heat-treated in a second step with a temperature gradient of 300 °C/h, keeping the samples at 600 °C, 700 °C, 800 °C, 900 °C, and 1000 °C, each for 1 h.

The changes in the density, porosity, and specific surface area (BET) of the silica matrix were tracked in annealed aerogels using nitrogen adsorption-desorption porosimetry. The size and shape of the gold nanoparticles in solutions and the PVP-stabilized and non-stabilized aerogels were checked using transmission electron microscopy (TEM). Scanning electron microscopy (SEM) and high-power 3D optical microscopy were used to explore the structure and morphology of the aerogel samples.

### 4.4. Catalytic Studies

The catalytic activity of the gold nanoparticles was studied by reducing 4-nitrophenol with sodium borohydride, using both colloidal AuNP solutions and their embedded forms in silica aerogels. A reaction mixture was prepared in a quartz cuvette by adding 60 µL of 4-nitrophenol aqueous solution (7.12 × 10^−^⁴ mol/dm^3^) followed by 0.50 mL of NaBH_4_ solution (2.64 × 10^−2^ mol/dm^3^). The reaction was conducted at the natural pH of NaBH_4_ at 25 °C with consistent experimental conditions across tests. The cuvette was equipped with dual stirring (top and bottom) and an argon gas inlet to remove bubbles from the light path and maintain an inert atmosphere. The absorbance values of the solutions were recorded continuously as a function of the reaction time.

Heterogeneous catalytic reactions using PVP-stabilized and non-stabilized aerogels were carried out with the same reagent solutions in 10.00 mL batch volumes using four times the reagent volumes and either 84 mg or 60 mg of the selected aerogels. The solutions were stirred under an argon atmosphere, with samples for UV–vis photometric measurements regularly taken and returned to the batch after analysis. Continuous flushing of the quartz cuvette with argon gas ensured isolation from atmospheric oxygen. A variable-speed magnetic microstirrer was used to eliminate hydrogen bubbles, either at the top or bottom of the cuvette, preventing interference with the absorbance measurements. Before performing the “live” kinetic measurements with nanogold-containing aerogels, an identical amount of zero-gold “blank” silica aerogel (prepared via the same method and heat treated at 500 °C for 5 h) was tested and found to be catalytically inactive.

## Figures and Tables

**Table 1:** Porosimetry studies of PVP-stabilized and reverse-engineered gold nanoparticle-containing aerogels.

PVP-stabilized samples	SH27	SH28	SH29	SH30	SH31	SH32
	500 °C	600 °C	700 °C	800 °C	900 °C	1000 °C
BET area (m^2^/g)	890	762	720	461	306	<20
Characteristic pore diameter (nm)	20	24	24	20	17	3
Reverse-engineered samples	SH45	SH46	SH47	SH48	SH49	SH50
	500 °C	600 °C	700 °C	800 °C	900 °C	1000 °C
BET area (m^2^/g)	899	790	702	625	458	<20
Characteristic pore diameter (nm)	17	20	20	17	13	3

**Table 2:** Apparent first-order reaction rate constants (k) and half-lives (t_1/2_) of nanogold solution and the aerogel catalysts containing the same amount of gold dispersed in the silica aerogel matrix.

Catalyst	Amount of Catalyst	k (min^−1^)	t_1/2_ (min)
Colloidical gold nanoparticles	10 µL	0.104	6.66
500 °C (PVP)	60.2 mg	0.099	7.00
600 °C (PVP)	60.0 mg	0.141	4.92
700 °C (PVP)	60.3 mg	0.094	7.37
800 °C (PVP)	60.1 mg	0.107	6.48
500 °C (rev)	84.0 mg	0.076	9.12

## Data Availability

Data are contained within the article.
